# Achieving 100% Uptake of Intravenous Zoledronic Acid After Hip Fracture Through Rapid Vitamin D Loading: A Three-Cycle Quality Improvement Study in a District General Hospital

**DOI:** 10.7759/cureus.98225

**Published:** 2025-12-01

**Authors:** Zarah Wilson, Zain Habib, Geraint Williams, Azhar Saeed, Sadhika Vinod, Mahwish Naureen

**Affiliations:** 1 Internal Medicine, North Manchester General Hospital, Manchester, GBR; 2 Trauma and Orthopaedics, Manchester Foundation Trust, Manchester, GBR; 3 Orthopaedics, North Manchester General Hospital, Manchester, GBR; 4 Trauma and Orthopaedics, North Manchester General Hospital, Manchester, GBR; 5 General Medicine, Manchester Foundation Trust, Manchester, GBR

**Keywords:** fragility hip fracture, nhfd, osteoporosis, secondary fracture prevention, zoledronic

## Abstract

Introduction: Hip fracture patients are at high risk of secondary fractures. National guidance and the consensus from the literature recommend timely bone protection therapy. Delays in vitamin D testing and logistical barriers often prevent inpatient intravenous (IV) zoledronic acid use. Our aim was to determine whether a rapid vitamin D loading protocol enabling inpatient IV zoledronic acid administration before discharge could improve treatment uptake and meet national standards.

Methods: A three-cycle quality improvement project was conducted in patients aged ≥60 years with fragility hip fractures. Baseline data (2020-2022) were compared to post-intervention cycles (2024-2025). The intervention comprised a standard operating procedure (SOP) incorporating ≥160,000 IU cholecalciferol loading over seven days, renal and calcium monitoring, pre- and post-infusion checklists, and inpatient IV zoledronic acid on or after day 5 post-surgery. The primary outcome was receipt of IV zoledronic acid. Secondary measures included vitamin D result availability, process compliance, and safety outcomes.

Results: At baseline, seven out of 29 patients (24.1%) received IV zoledronic acid. Post-intervention, all eligible patients (100%) (Cycle 2: 30/30; Cycle 3: 80/80) received inpatient IV zoledronic acid (*p*<0.001). Vitamin D results were unavailable at infusion in 53 patients (66.2%) of Cycle 3 patients, yet 80 patients (100%) completed anticipatory loading and 80 patients (100%) had calcium checks. No hypocalcaemia or infusion-related complications occurred. Six-month re-fracture and mortality rates were 1.25% (one patient) and 6.3% (five patients), respectively. National Hip Fracture Database (NHFD) Key Performance Indicator 7 (KPI-7)compliance reached 100%.

Conclusion: A rapid vitamin D loading SOP enabled timely inpatient IV zoledronic acid administration, overcoming logistical and laboratory barriers and achieving 100% compliance with national standards without adverse effects. This pathway is safe, sustainable, and scalable for secondary fracture prevention in district general hospitals.

## Introduction

Fragility hip fractures are associated with high morbidity, mortality, and healthcare costs in older adults. One-year mortality rates range from 20-30%, and the risk of subsequent fractures is substantial [1,2]. National and international guidance strongly emphasises secondary fracture prevention following hip fracture. The National Institute for Health and Care Excellence (NICE) Clinical Guideline 146 (CG146) recommends assessing fracture risk and initiating bone-protection therapy in all patients with fragility fractures. NICE Technology Appraisal 464 (TA464) specifically recommends bisphosphonates, including intravenous zoledronic acid, as cost-effective first-line treatment for secondary prevention, noting improved adherence compared with oral therapy [3,4].

The National Osteoporosis Guideline Group (NOGG) 2024 guideline further advises that intravenous zoledronic acid should be used as first-line therapy after hip fracture because it reduces refracture risk and mortality and has superior adherence relative to oral bisphosphonates [5]. In parallel, the National Hip Fracture Database (NHFD) Key Performance Indicator 7 (KPI-7) mandates that bone protection medication must be initiated or supported within 120 days of hip fracture as a national quality standard [6]. Denosumab was not selected as first-line therapy because NOGG and NICE recommend intravenous zoledronic acid as the preferred treatment following hip fracture, owing to its demonstrated mortality benefit, superior long-term adherence, and suitability for single-dose inpatient administration. Denosumab is reserved for patients who cannot receive bisphosphonates, which did not apply to the eligible cohort [4,5].

Despite this robust evidence and policy framework, national treatment rates remain suboptimal at approximately 50-60% by 120 days post-fracture [1,6]. A major real-world barrier is the perceived need to confirm vitamin D sufficiency before intravenous (IV) bisphosphonate administration, leading to delays, outpatient non-attendance, and treatment omission. Additionally, adherence to oral bisphosphonates is poor [4,7]. In 2024, our institution implemented a rapid vitamin D loading standard operating procedure (SOP) with checklist-based safety monitoring and inpatient IV zoledronic acid administration on or after postoperative day 5. This approach aimed to eliminate delays, improve uptake, and align care with national standards. We aimed to evaluate whether a rapid vitamin D loading SOP could achieve near-universal inpatient IV zoledronic acid administration and sustained compliance with NHFD KPI-7 in a district general hospital.

## Materials and methods

A three-cycle quality improvement (QIP) project was conducted at a district general hospital to evaluate and improve intravenous zoledronic acid use after hip fracture. Cycle 1 (2020-2022) served as the pre-intervention baseline. Following the implementation of a new bone health protocol in April 2024, Cycle 2 assessed immediate post-intervention outcomes, and Cycle 3 (2024-2025) evaluated sustainability. The project followed the Plan-Do-Study-Act methodology. Clinical judgment remained the primary determinant in establishing a diagnosis of osteoporosis among patients presenting with a fragility hip fracture, particularly where recognised risk factors were evident. Bone mineral density (BMD) measurements were not considered a limiting factor for the initiation of osteoporosis treatment in such cases, as the presence of a fragility fracture in a clinically at-risk individual was deemed sufficient to warrant therapy.

Given the practical limitations within a District General Hospital (DGH) setting, the absence of immediate or contemporaneous dual-energy X-ray absorptiometry (DEXA) results during the acute inpatient phase, following hip fracture-eligible patients were commenced on parenteral osteoporosis therapy during their admission. Post-discharge, DEXA scanning was organised as part of a structured outpatient follow-up to facilitate ongoing evaluation within the established osteoporosis referral pathway. Patients identified as requiring further assessment or deemed suitable for anabolic therapy were subsequently referred to Rheumatology services within Manchester Foundation Trust (MFT), in line with institutional clinical governance protocols and national best practice standards. 

Although DEXA scanning can support longer-term treatment planning, it is not required before initiating therapy in patients presenting with a fragility hip fracture. In our setting, inpatient DEXA is not available, and waiting for imaging would delay treatment during a period when early intervention is important. To maintain timely care while still allowing appropriate evaluation, patients who may require further stratification were referred for post-discharge DEXA and specialist review. This approach ensured safe inpatient administration of zoledronic acid without compromising downstream assessment or treatment selection.

Eligible participants were consecutive patients aged ≥60 years admitted with fragility hip fractures and had a clinical recommendation - indication for intravenous zoledronic acid following specialist multidisciplinary assessment. Data were obtained from electronic health records and orthopaedic databases. After data cleaning, sample sizes were: Cycle 1 (n=30), Cycle 2 (n=30), and Cycle 3 (n=80); 80 patients in Cycle 3 were eligible for zoledronic acid. The intervention was a Standard Operating Procedure (SOP) that enabled inpatient zoledronic acid administration prior to discharge. It included: (1) rapid vitamin D loading (≥160,000 IU cholecalciferol over ~7 days), (2) mandatory pre-infusion safety checks (renal function and adjusted serum calcium within 72 hours) supported by pre- and post-infusion checklists and GP notification, and (3) intravenous zoledronic acid 5 mg administered on or after postoperative day 5.

Expert group consensus advice was followed, which stated that vitamin D deficiency is prevalent among patients presenting with hip fractures. In cases where vitamin D status is unknown but serum calcium concentration is within the normal reference range, the therapeutic advantages of administering an empirical high-dose vitamin D loading regimen outweigh the potential risks. This approach facilitates the prompt initiation of intravenous zoledronic acid during the inpatient episode. Appropriate oral loading regimens may comprise cumulative doses between 150,000 and 250,000 international units administered over one to seven days.

The expert consensus group recommended a maximum cumulative loading dose of 250,000 international units (IU) of vitamin D, with a minimum effective dose of 150,000 IU. Accordingly, during the development of this SOP, a total dose of 150,000 IU was adopted as the baseline regimen. To achieve this, a divided dosing schedule of 40,000 IU daily for seven consecutive days was established. This regimen ensures that a minimum cumulative dose of 160,000 IU is attained by day four, thereby providing adequate vitamin D repletion to safely permit the administration of intravenous zoledronic acid on day five postoperatively. A minimum total dose of 160,000 international units was determined to represent a clinically acceptable and safe threshold for proceeding with intravenous zoledronic acid therapy. This strategy optimises timely treatment delivery while concurrently mitigating the risk of hypocalcaemia in individuals naïve to vitamin D supplementation. This approach is consistent with national expert consensus guidance from the Royal Osteoporosis Society, which supports empirical high-dose vitamin D loading in patients with fragility fractures when vitamin D status is unknown, and serum calcium is normal [8].

The primary outcome was the proportion of eligible patients receiving intravenous zoledronic acid. Secondary process measures included vitamin D result availability at infusion, completion of loading, checklist adherence, calcium monitoring, and GP notification. Clinical outcomes in Cycle 3 included six-month refracture, mortality, and ASA (American Society of Anesthesiologists) physical status classification. Cycle 1 data were retrospective; Cycles 2 and 3 were collected prospectively using a standardised proforma. Categorical data were summarised as proportions. Fisher’s exact test compared zoledronic acid administration between cycles, and relative risks (RR) with 95% confidence intervals (CI) were calculated. Relative risks (RR) with 95% confidence intervals (CI) were calculated to compare post-intervention cycles (Cycle 2 and Cycle 3) with baseline (Cycle 1). Statistical significance was defined as p<0.05. In Cycle 3, 80/80 eligible patients had documented administration; five had incomplete documentation despite evidence of pathway progression. This project was registered with the hospital audit department. According to local policy, formal ethics approval was not required for this anonymised service evaluation. The vitamin D loading regimen formed part of a Trust-ratified clinical standard procedure and was delivered as routine care; therefore, no additional ethics approval was required.

## Results

A total of 145 patients were included: 29 in Cycle 1, 30 in Cycle 2, and 86 in Cycle 3. In Cycle 3, 80 patients were eligible for IV zoledronic acid. The mean ASA score in Cycle 3 was 3.06 (median 3), indicating significant baseline frailty. Data on IV bisphosphonate administration is depicted below in Table 1.

**Table 1:** IV zoledronic acid administration between audit cycles IV: Intravenous.

Comparison	Baseline Zoledronic Acid Administration Cycle 1 (n/%)	Post-Intervention Zoledronic Acid Administration (Cycle 2/3) (n/%)	Relative Risk (RR)	95% Confidence Interval (CI)	p-value
Cycle 2 vs Cycle 1	7/30 (23.3%)	30/30 (100%)	4.14	2.17 – 7.90	<0.001
Cycle 3 vs Cycle 1	7/30 (23.3%)	80/80 (100%)	4.14	2.17 – 7.90	<0.001

IV zoledronic acid administration increased from seven out of 30 patients (23.3%) at baseline to 80 out of 80 patients (100%) post-intervention (p<0.001), sustained one year later. Compared with baseline, the likelihood of receiving IV zoledronic acid was over four times higher in both Cycle 2 and Cycle 3 (RR 4.14, 95% CI 2.17-7.90). Secondary outcomes included salient variables from the process developed and clinical outcomes from Cycle 3 are depicted below in Tables 2, 3.

**Table 2:** Process measures

Process Measures	Frequency (n/%)
Vitamin D result available at fusion	27/80 (33.8%)
Vitamin D loading ≥ 160,000 IU	80/80 (100%)
Pre-infusion checklist performed	79/80 (98.8%)
Calcium checked ≤ 72 hours pre-infusion	80/80 (100%)
Post-infusion checklist completed	71/80 (88.8%)
GP notification documented	79/80 (98.8%)

**Table 3:** Clinical outcomes for Cycle 3 within six months IV: Intravenous.

Outcome	Frequency (n/%)
Re-fracture within six months in 30 of the 80 patient cohort, who had IV zoledronic acid minimum six months ago	1/30 of 80 (1.25%)
Mortality within six months	5/80 (6.3%)

Despite limited vitamin D result availability, high adherence to anticipatory loading and safety monitoring ensured timely inpatient therapy. Early inpatient IV zoledronic acid was well tolerated and associated with low re-fracture and acceptable mortality rates. Treatment compliance remained 100% in Cycle 3, demonstrating sustained improvement.

## Discussion

This project demonstrates that a rapid vitamin D loading SOP embedded within routine hip fracture care can achieve and sustain universal inpatient IV zoledronic acid administration, effectively bridging the gap between national evidence-based recommendations and real-world practice. The findings align with the HORIZON Recurrent Fracture Trial, where IV zoledronic acid initiated within 90 days post-hip fracture reduced refracture and mortality [9]. These results directly support the NICE TA464 recommendation to use IV bisphosphonates as first-line therapy after fragility fractures [4] and the NOGG 2024 guideline, which specifically endorses IV zoledronic acid after hip fracture due to its superior adherence and efficacy [5].

This pathway also meets the National Hip Fracture Database (NHFD) Key Performance Indicator 7 (KPI-7), which mandates initiation or support of bone protection treatment within 120 days [6]. Nationally, KPI-7 compliance is typically around 50-60% [1]. In contrast, this intervention achieved 100% compliance, demonstrating that inpatient delivery overcomes outpatient attrition and poor adherence to oral therapy [9-12]. Safety was ensured through mandated calcium monitoring and checklist-based processes. Despite 67.5% not having vitamin D results at infusion, anticipatory loading (≥160,000 IU cholecalciferol) was completed in 98.8%, and no cases of hypocalcaemia occurred, consistent with UK guidance that such loading regimens are safe and appropriate to facilitate timely bisphosphonate therapy [8,13].

Over a period of six months to one year, the proposed Standard Operating Procedure (SOP) underwent a rigorous governance review and approval process, including iterative evaluation and consultation with the Medicines Management Committee (MMC) at Manchester Foundation Trust (MFT) and the institutional governance team. This comprehensive review culminated in the electronic verification and ratification of the policy, now formally accessible via the MFT intranet. In April 2024, the MMC granted formal ratification of the SOP, with a prospective revalidation date set for April 2027. The approved framework authorises anticipatory vitamin D loading followed by intravenous zoledronic acid administration for eligible inpatients prior to hospital discharge. Low six-month refracture (1.25%) and mortality (6.3%) rates support the clinical value of early IV zoledronic acid, aligning with published data [2]. The sustainability of 100% uptake one year post-implementation highlights the effectiveness of embedding the SOP into orthogeriatric workflows with multidisciplinary support and Medicines Management Committee ratification.

Strengths of this study include real-world implementation, clear process documentation, and sustained improvement. Limitations include retrospective baseline data, minor documentation gaps, a single-centre design, and a six-month follow-up. Future work should assess longer-term outcomes, patient-reported measures, and cost-effectiveness, and explore application to other fragility fractures within fracture liaison services. This pragmatic, low-cost inpatient pathway demonstrates how hospitals can operationalise NICE, NOGG, and NHFD standards, delivering high-value care and improving secondary fracture prevention.

From a health-economics perspective, the potential fiscal benefit of timely inpatient delivery of IV zoledronic acid is substantial. In England and Wales, the mean inpatient cost in the year after a hip fracture is approximately £14,465 per patient, with nearly £12,547 of that incurred in the first 120 days alone. Even modest reductions in refracture incidence therefore translate into meaningful savings for the NHS [14]. Zoledronic acid infusions have also been found to be fiscally more effective than sequential denosumab and alendronate used in other centres [15]. Looking ahead, the prospective development plan aims to expand the patient cohort and incorporate a control group to strengthen outcome analysis. The clinical impact and effectiveness of this policy will be evaluated prospectively, with a particular focus on the incidence of subsequent fragility fractures within one year post-infusion. Data collection and outcome monitoring will be facilitated through the existing Bone Health Clinic database, coordinated by the Orthogeriatric team, to ensure systematic evaluation and ongoing quality improvement in secondary fracture prevention.

## Conclusions

A rapid vitamin D loading SOP enabled safe, universal inpatient IV zoledronic acid administration after hip fracture, achieving 100% treatment uptake, meeting NHFD KPI-7, and sustaining performance over one year. This model is practical, scalable, and aligned with national guidelines, offering a robust approach to improving secondary fracture prevention in district general hospitals.

## Figures and Tables

**Figure 1:**
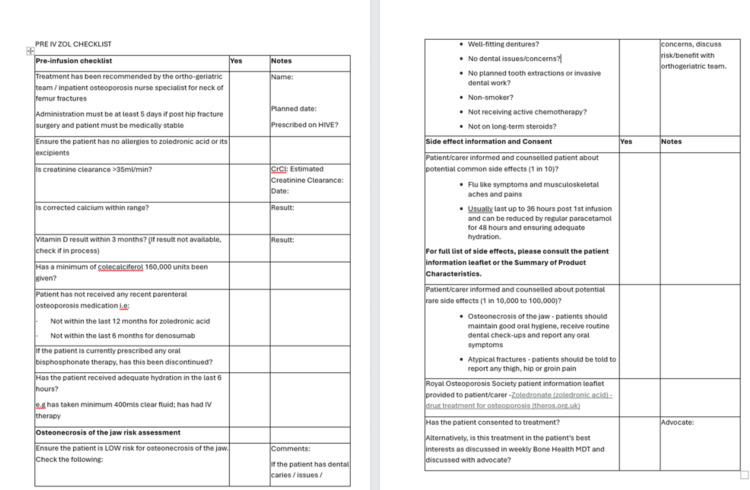
Pre IV zoledronic acid administration checklist IV: Intravenous.
